# Wealth‐Based Inequalities in Child Undernutrition and the Double Burden of Malnutrition Among Child‐Mother Dyads at the Same Household in Bangladesh: A Decomposition Approach Using Cross‐Sectional Data

**DOI:** 10.1002/hsr2.71628

**Published:** 2025-12-11

**Authors:** Sumaya Sultana, Khondokar Naymul Islam, Syed Afroz Keramat, Benojir Ahammed

**Affiliations:** ^1^ Department of Statistics, Faculty of Science Pirojpur Science and Technology University Pirojpur Bangladesh; ^2^ Statistics Discipline, Science, Engineering and Technology School Khulna University Khulna Bangladesh; ^3^ Centre for Health Services Research, Faculty of Health, Medicine and Behavioural Sciences The University of Queensland Brisbane Queensland Australia; ^4^ School of Business and Centre for Health Research University of Southern Queensland Toowoomba Australia

**Keywords:** child undernutrition, concentration index, decomposition, double burden of malnutrition, inequalities

## Abstract

**Backgrounds and Aims:**

Child undernutrition and the double burden of malnutrition (DBM) at the household level are pressing public health issues. Still, an understanding of the wealth‐based inequalities in child undernutrition and DBM among child‐mother dyads has received little attention, particularly in Bangladesh. This study examines wealth‐based inequality, the associated factors of child malnutrition, and DBM.

**Methods:**

This study analysed data from the most recent Bangladesh Demographic and Health Survey (BDHS) conducted in 2017–18. The DBM refers to the coexistence of an undernourished child and an overweight/obese mother (body mass index ≥ 25 kg/m²) within the same household. This study employed a binary logistic regression model to identify the characteristics associated with undernutrition in children and DBM. Additionally, concentration curve and index were utilized to assess wealth‐based inequalities, and regression‐based decomposition was applied to explore their underlying causes in child undernutrition and DBM.

**Results:**

This study found that 38.54% of children experienced undernutrition, and DBM impacted 7.50% of child‐mother pairs. The concentration index for child undernutrition and the DBM were −0.191 (pro‐poor inequality) and 0.163 (pro‐rich inequality), respectively. The decomposition analysis indicated that household wealth status, maternal education, and paternal education accounted for 41.34%, 15.54%, and 14.33%, respectively, of socio‐economic inequality in child undernutrition. Conversely, their contributions to socio‐economic inequality in DBM were 93.49%, −32.05%, and 15.20%.

**Conclusion:**

Health policymakers and authorities must prioritise factors such as wealth, education, and living conditions to reduce child undernutrition and DBM in Bangladesh. Prompt and decisive action is necessary to identify and address these issues effectively.

## 1 Introduction

Childhood undernutrition is a critical global public health issue that manifests as stunting, wasting, and being underweight, while overnutrition leads to overweight and obesity. Physical signs of malnutrition can often be identified without special tools; however, some forms remain challenging to detect. Beyond the visible signs, malnutrition often takes less apparent forms that are harder to notice [1]. Reducing malnutrition is vital for achieving sustainable development goals (SDGs) allied to food security, hunger, poverty, sustainable agriculture, and health. Addressing child malnutrition is crucial as it impacts early cognitive development and highlights the importance of tackling social determinants of health for overall well‐being [2, 3, 4]. Ensuring children's health is fundamental to meeting SDG 3.2, which seeks to decrease under‐five mortality to 2.5% of live births by 2030 [5]. While the SDGs prioritise reducing health inequalities, comprehensive equity analysis of childhood undernutrition in Bangladesh remains limited, with most studies focusing solely on stunting [6, 7].

A growing prevalence of overweight and obesity among women of reproductive age has been noted in Central and Southeast Asia, with significantly higher rates observed among women from wealthier households compared to their less affluent counterparts [8]. A study conducted across South Asian countries found a significant positive association between household wealth and the risk of overweight and obesity, suggesting that individuals from wealthier households are more likely to experience excess body weight [9, 10]. In South Asia, including Bangladesh, the increased prevalence of the overweight burden among women is directly linked to higher socioeconomic status, even after adjusting for other influencing factors [8]. Research has consistently shown that excess body weight significantly increases the risk of mortality [11], cardiovascular diseases [12], diabetes [13], various other health conditions [12, 14], and disability [15]. A projections indicate that by 2030, the incidence of overweight and obesity conditions associated with adverse maternal and fetal health outcomes will rise by approximately two‐thirds in South and Southeast Asia [16].

Economic status significantly contributes to undernutrition, as low‐income households often lack access to sufficient, diverse, and nutritious food, leading to inadequate dietary intake [17]. Additionally, poverty limits access to healthcare, sanitation, and education, which are critical factors in preventing and managing undernutrition [18]. Economic growth often leads to increased disposable income and urbanization, which can promote sedentary lifestyles and greater consumption of energy‐dense, processed foods, contributing to being overweight and obesity [19]. Additionally, as countries develop, traditional diets and physical labour are often replaced by high‐calorie diets and reduced physical activity, fueling the obesity epidemic [20].

The double burden of malnutrition (DBM) occurs when a mother is overweight or obese while a child in the same household experiences undernutrition [21], posing risks of childhood mortality and cognitive impairment [22], and maternal diseases like hypertension and diabetes [23]. The simultaneous burden of maternal overweight or obesity and child undernutrition is driven by socioeconomic conditions, dietary habits, and physical activity levels [24], and is further influenced by factors such as maternal education, age, and household wealth [25, 26]. Additional factors including the child's age [6, 27, 28, 29] and sex [27, 28, 29], birth order [6, 27, 28, 30, 31], paternal education [6, 28, 30], place of residence [1, 29, 31], geographic region [1, 6, 30, 31], source of drinking water [28, 31], type of sanitation facility [6, 27, 28, 29], and cooking fuel [28] have also been identified in previous studies as being associated with the DBM or its individual components.

Nutrition transition is often characterised by the concurrent rise of undernutrition and overnutrition [12]. The growing recognition of wealth‐based disparities in childhood undernutrition is spurring research and policy initiatives, resulting in a broader body of health equality literature [32]. Assessing the extent of inequality in undernutrition, such as stunting, wasting, and being underweight, across wealth groups is essential, as is identifying the key factors that contribute to these disparities. Economic progress and improved socio‐economic conditions may reduce undernutrition, but they also contribute to a growing trend of overweight and obesity. To advance equality, a core principle of the SDGs, it is vital to understand wealth‐based malnutrition disparities in Bangladesh.

In Asia, particularly in Bangladesh, the DBM is an escalating concern, with both obesity and undernutrition becoming increasingly prevalent [33]. Several prior studies have documented the prevalence and determinants of child undernutrition and maternal overweight or obesity in Bangladesh [34, 35, 36]. Limited research has explored the coexistence of these conditions commonly referred to as DBM within the same household, particularly among child‐mother pairs. Moreover, although socioeconomic disparities are recognised as key drivers of malnutrition [10], there is a lack of in‐depth analysis that quantifies the contribution of wealth‐based inequalities to this dual burden. Most existing studies fail to apply decomposition techniques that can disentangle the specific socioeconomic and demographic factors contributing to such inequalities [6, 37]. Therefore, a comprehensive examination using a decomposition approach is essential to better understand the structural sources of child undernutrition and DBM and to inform targeted, equity‐sensitive interventions.

This study examines undernutrition and the DBM in Bangladesh child‐mother pairs, exploring the coexistence of both issues within households, while addressing contributing factors and wealth‐based inequalities. It thereby fills a critical gap in understanding health disparities in a country with uneven progress in health and development.

## 2 Materials and Methods

### 2.1 Data Sources and Study Design

**Figure 1:**
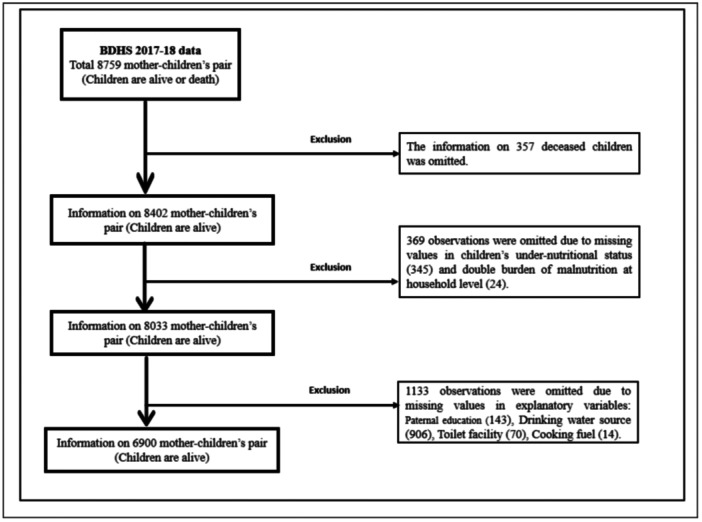
Data selection criteria.

The data for this study were sourced from the 2017–18 Bangladesh Demographic and Health Survey (BDHS), implemented by the National Institute of Population Research and Training (NIPORT) under the Ministry of Health and Family Welfare, in partnership with Mitra and Associates. Employing a two‐stage stratified random sampling technique, the survey selected 672 enumeration areas (249 urban and 423 rural) and, within each area, systematically sampled 30 households, resulting in a final total of 20,160 households [38]. This study was restricted to ever‐married women aged 15–49 years with at least one child under 5 years of age residing in the same household. The study examined the prevalence and determinants of the DBM at the household level and childhood undernutrition in Bangladesh. To prepare the analysable dataset, 1859 cases (21.22%) from a total of 8759 child–mother pairs were omitted due to missing or ineligible information. Specifically, the exclusions included 357 (4.08%) children who were no longer alive, 345 (3.94%) cases with missing data on child undernutrition, 24 (0.27%) on maternal overweight/obesity, 143 (1.63%) on paternal education, 906 (10.35%) on source of drinking water, 70 (0.79%) on toilet facility, and 14 (0.16%) on cooking fuel. Consequently, a total of 6900 child–mother pairs were retained for the final analysis. Figure 1 outlines the final sample selection process.

### 2.2 Outcome Variables

This study focused on two main outcome variables: children's nutritional status and the DBM at the household level, both of which were binary. Children were classified as stunted, wasted, or underweight based on WHO criteria, with Z‐scores bellow ‐2 SD from the reference population median for height‐for‐age, weight‐for‐height, and weight‐for‐age, respectively [39]. Children were classified as undernourished (1) if they were stunted, wasted, or underweight, and as not undernourished (0) otherwise. The second outcome variable, the DBM at the household level, was defined as the coexistence of overnutrition (overweight or obesity in mother) and undernutrition (stunting, wasting, or underweight in children) within the same household. Women's nutritional status was measured using BMI, with overweight or obese defined as BMI ≥ 25.0 kg/m² [40], and the DBM was categorised as 1 when both an overweight or obese mother and an undernourished child were in the same household, and otherwise was categorized as 0 [27].

### 2.3 Explanatory Variables

This study included 14 explanatory variables pertaining to children's, parental, and household characteristics, chosen based on existing scholarly literature and the availability of data [6, 27, 28, 29, 41, 42, 43, 44]. These variables include child‐related factors: age ( < 12, 12–23, 24–35, 36–47, 48–59 months), sex (male, female), and birth order (1, 2–4, ≥ 5); parental characteristics: maternal age (15–24, 25–34, 35–49 years), maternal education (no education, primary, secondary, higher), paternal education (no education, primary, secondary, higher), age at first birth ( > 18, ≤ 18 years), and media exposure (yes, no); and household factors: residence (rural, urban), wealth index (poorest, poorer, middle, richer, richest), the administrative structure of Bangladesh, known as division (Barisal, Chittagong, Dhaka, Khulna, Mymensingh, Rajshahi, Rangpur, Sylhet), source of drinking water (improved, unimproved), toilet facility (improved, unimproved), and cooking fuel (improved, unimproved). Media exposure was defined by a woman's weekly use of television, radio, or newspapers [27]. Drinking water sources were categorised as improved or unimproved, with improved sources including piped water, public taps, standpipes, tube wells, boreholes, protected dug wells or springs, rainwater, water delivered via a tanker truck or cart with a small tank, and bottled water. Toilet facilities were categorized as improved (e.g., flush systems pit latrines with slab or ventilation) or unimproved (e.g., field, hanging or bucket toilets), and cooking fuel was divided into clean (e.g., electricity, liquid petroleum gas/natural gas/biogas dung) and unclean (e.g., coal, wood charcoal, straw/shrubs/grass, agricultural crops, and animals) categories [38].

### 2.4 Statistical Analysis

This study employed various statistical methods to analyse the data. All statistical analyses were conducted using weighted data to ensure that the results are representative at the national level. Firstly, frequency distribution and percentage reporting of baseline variables were utilised. The weighted prevalence of child undernutrition and the DBM at the household level was computed with a 95% confidence interval (CI). The chi‐square test was used to explore bivariate relationships between outcome and explanatory variables, while binary logistic regression determined the impact of explanatory factors on these outcomes, reporting adjusted odds ratios (AOR) with 95% CI. A p‐value below 0.05 was interpreted as evidence of statistical significance in a two‐sided test. In examining wealth‐based inequalities in nutritional status of children and the DBM, a concentration curve, concentration index (CIX), and CIX decomposition were applied. The socio‐economic disparities were assessed by plotting the cumulative percentage of an event against the wealth index [30]. Statistical analyses were carried out using STATA 17 (MP) software.

The CIX quantifies wealth‐related disparities, with values ranging from −1 to +1, where 0 represents no socio‐economic disparity. Negative and positive CIX values indicate inequality among lower or higher wealth quintiles, respectively [45]. The CIX value can be calculated using the following equation:

$$\mathrm{CIX}=\frac{2}{\mu }\mathrm{cov}\left(y,r\right)$$

where: CIX= concentration index; µ= weighted mean of children's undernutrition and the DBM; y= outcome variable; r= fractional rank of the individual in the distribution of wealth index; cov = weighted covariance between y and r.

The relative CIX can be decomposed to ascertain the contribution of each explanatory variable to the overall wealth‐associated inequality, employing methods proposed by World Bank Group [45]. The contribution of every single variable is determined by multiplying its wealth‐related inequality degree by its elasticity, with the remaining unexplained inequality referred to as the residual.

## 3 Results

### 3.1 Baseline Characteristics

Table 1 presents the standard physical characteristics of the 6900 child‐mother pairs, with 52.25% of children being male and 58.36% having a birth order of 2–4. Nearly half of the mothers (47%) had secondary education, while the largest proportion of fathers (35.78%) had completed primary school. The majority of respondents lived in rural areas (65.01%). Most households used improved drinking water sources (97.99%) and had improved toilet facilities (64.97%), although 79.94% relied on unsafe cooking fuel.

**Table 1:** Baseline characteristic of considered variables, weighted prevalence and bivariate association of explanatory variables with child undernutrition and DBMHL.

Factor	Overall, *n* (%)	Child undernutrition			DBMHL		
		No, *n* (%)	Yes, *n* (%)	Weighted prevalence,% (95% CI)	No, *n* (%)	Yes, *n* (%)	Weighted prevalence,% (95% CI)
**Overall**	6900 (100)	4192 (60.75)	2708 (39.25)	38.54 (37.39–39.70)	6385 (92.54)	515 (7.46)	7.50 (6.90–8.15)
**Children characteristics**							
**Age of child (in months)**		***p value*** =< ***0.001***			***p value*** =< ***0.001***		
< 12	1420 (20.58)	998 (70.28)	422 (29.72)	28.73 (26.42–31.16)	1357 (95.56)	63 (4.44)	4.19 (3.26–5.37)
12–23	1408 (20.41)	818 (58.10)	590 (41.90)	40.60 (38.07–43.17)	1327 (94.25)	81 (5.75)	5.71 (4.61–7.04)
24–35	1372 (19.88)	754 (54.96)	618 (45.04)	44.99 (42.37–47.63)	1239 (90.31)	133 (9.69)	9.43 (7.99–11.09)
36–47	1291 (18.71)	748 (57.94)	543 (42.06)	41.09 (38.45–43.79)	1165 (90.24)	126 (9.76)	10.15 (8.63–11.91)
48–59	1409 (20.42)	874 (62.03)	535 (37.97)	37.55 (35.01–40.16)	1297 (92.05)	112 (7.95)	8.32 (6.96–9.91)
**Sex of child**		***p‐value*** = ***0.580***			***p‐value*** = ***0.320***		
Male	3605 (52.25)	2179 (60.44)	1426 (39.56)	38.80 (37.21–40.41)	3325 (92.23)	280 (7.77)	7.71 (6.88–8.63)
Female	3295 (47.75)	2013 (61.09)	1282 (38.91)	38.25 (36.60–39.93)	3060 (92.87)	235 (7.13)	7.28 (6.43–8.22)
**Birth order**		***p‐value*** =< ***0.001***			***p‐value*** =< ***0.001***		
1	2469 (35.78)	1578 (63.91)	891 (36.09)	36.12 (34.24–38.04)	2331 (94.41)	138 (5.59)	5.96 (5.08–6.97)
2–4	4027 (58.36)	2422 (60.14)	1605 (39.86)	38.89 (37.40–40.41)	3687 (91.56)	340 (8.44)	8.31 (7.50–9.21)
≥ 5	404 (5.86)	192 (47.52)	212 (52.48)	49.89 (44.98–54.80)	367 (90.84)	37 (9.16)	8.84 (6.41–12.07)
**Parental characteristics**							
**Maternal age (in years)**		***p‐value*** = ***0.500***			***p‐value*** =< ***0.001***		
15–24	3080 (44.64)	1890 (61.36)	1190 (38.64)	38.30 (36.60–40.03)	2930 (95.13)	150 (4.87)	5.04 (4.32–5.87)
25–34	3218 (46.64)	1947 (60.50)	1271 (39.50)	38.58 (36.91–40.28)	2927 (90.96)	291 (9.04)	9.03 (8.09–10.07)
35–49	602 (8.72)	355 (58.97)	247 (41.03)	39.54 (35.62–43.59)	528 (87.71)	74 (12.29)	12.18 (9.75–15.11)
**Maternal education**		***p‐value*** =< ***0.001***			***p‐value*** = ***0.730***		
No education	510 (7.39)	234 (45.88)	276 (54.12)	52.07 (47.70–56.40)	474 (92.94)	36 (7.06)	7.00 (5.07–9.58)
Primary	2040 (29.57)	1088 (53.33)	952 (46.67)	45.73 (43.57–47.92)	1892 (92.75)	148 (7.25)	8.05 (6.94–9.32)
Secondary	3244 (47.01)	2010 (61.96)	1234 (38.04)	37.10 (35.47–38.76)	2990 (92.17)	254 (7.83)	7.51 (6.66–8.45)
Higher	1106 (16.03)	860 (77.76)	246 (22.24)	22.20 (19.74–24.86)	1029 (93.04)	77 (6.96)	6.64 (5.26–8.35)
**Paternal education**		***p‐value*** =< ***0.001***			***p‐value*** = *0.220*		
No education	1041 (15.09)	512 (49.18)	529 (50.82)	50.29 (47.25–53.33)	973 (93.47)	68 (6.53)	6.41 (5.07–8.08)
Primary	2469 (35.78)	1350 (54.68)	1119 (45.32)	43.38 (41.44–45.33)	2296 (92.99)	173 (7.01)	6.81 (5.89–7.87)
Secondary	2179 (31.58)	1404 (64.43)	775 (35.57)	34.97 (33.01–36.98)	1998 (91.69)	181 (8.31)	8.64 (7.54–9.89)
Higher	1211 (17.55)	926 (76.47)	285 (23.53)	23.96 (21.55–26.55)	1118 (92.32)	93 (7.68)	7.78 (6.35–9.51)
**Maternal age at first birth**		***p‐value*** =< ***0.001***			***p‐value*** = *0.220*		
> 18	2917 (42.28)	1886 (64.66)	1031 (35.34)	34.81 (33.06–36.59)	2686 (92.08)	231 (7.92)	8.03 (7.08–9.10)
≤ 18	3983 (57.72)	2306 (57.90)	1677 (42.10)	41.11 (39.60–42.63)	3699 (92.87)	284 (7.13)	7.14 (6.38–7.97)
**Maternal media exposure**		***p‐value*** =< ***0.001***			***p‐value*** =< ***0.001***		
No	2585 (37.46)	1393 (53.89)	1192 (46.11)	45.11 (43.16–47.08)	2427 (93.89)	158 (6.11)	6.22 (5.33–7.24)
Yes	4315 (62.54)	2799 (64.87)	1516 (35.13)	34.82 (33.42–36.25)	3958 (91.73)	357 (8.27)	8.23 (7.45–9.08)
**Household characteristics**							
**Wealth index**		***p‐value*** =< ***0.001***			***p‐value*** =< ***0.001***		
Poorest	1533 (22.22)	767 (50.03)	766 (49.97)	49.01 (46.46–51.55)	1454 (94.85)	79 (5.15)	5.32 (4.28–6.58)
Poorer	1364 (19.77)	733 (53.74)	631 (46.26)	44.52 (41.91–47.16)	1291 (94.65)	73 (5.35)	5.03 (3.99–6.32)
Middle	1227 (17.78)	758 (61.78)	469 (38.22)	36.68 (34.09–39.34)	1133 (92.34)	94 (7.66)	7.41 (6.11–8.97)
Richer	1395 (20.22)	891 (63.87)	504 (36.13)	36.06 (33.59–38.62)	1257 (90.11)	138 (9.89)	10.22 (8.74–11.92)
Richest	1381 (20.01)	1043 (75.52)	338 (24.48)	24.91 (22.65–27.33)	1250 (90.51)	131 (9.49)	9.75 (8.25–11.47)
Residence		***p‐value*** =< ***0.001***			***p‐value*** =< ***0.001***		
Rural	4486 (65.01)	2605 (58.07)	1881 (41.93)	40.40 (39.05–41.78)	4188 (93.36)	298 (6.64)	6.69 (6.03–7.42)
Urban	2414 (34.99)	1587 (65.74)	827 (34.26)	33.60 (31.50–35.76)	2197 (91.01)	217 (8.99)	9.64 (8.39–11.06)
**Division**		***p‐value*** =< ***0.001***			***p‐value*** = ***0.027***		
Barisal	717 (10.39)	429 (59.83)	288 (40.17)	41.90 (37.05–46.91)	660 (92.05)	57 (7.95)	7.33 (5.11–10.41)
Chittagong	1088 (15.77)	658 (60.48)	430 (39.52)	39.66 (37.11–42.27)	991 (91.08)	97 (8.92)	8.72 (7.34–10.33)
Dhaka	1009 (14.62)	670 (66.40)	339 (33.60)	34.00 (31.82–36.25)	918 (90.98)	91 (9.02)	8.33 (7.12–9.71)
Khulna	741 (10.74)	517 (69.77)	224 (30.23)	31.24 (27.82–34.88)	696 (93.93)	45 (6.07)	5.66 (4.13–7.70)
Mymensingh	841 (12.19)	475 (56.48)	366 (43.52)	42.47 (38.57–46.46)	796 (94.65)	45 (5.35)	5.16 (3.66–7.25)
Rajshahi	718 (10.41)	448 (62.40)	270 (37.60)	39.43 (36.10–42.86)	662 (92.20)	56 (7.80)	8.41 (6.68–10.54)
Rangpur	745 (10.80)	476 (63.89)	269 (36.11)	37.02 (33.52–40.67)	690 (92.62)	55 (7.38)	6.57 (4.96–8.67)
Sylhet	1041 (15.09)	519 (49.86)	522 (50.14)	52.16 (48.09–56.20)	972 (93.37)	69 (6.63)	6.61 (4.86–8.94)
**Drinking water source**		***p‐value*** = ***0.007***			***p‐value*** = ***0.150***		
Improved	6761 (97.99)	4123 (60.98)	2638 (39.02)	38.36 (37.20–39.53)	6252 (92.47)	509 (7.53)	7.57 (6.96–8.23)
Unimproved	139 (2.01)	69 (49.64)	70 (50.36)	48.70 (39.82–57.67)	133 (95.68)	6 (4.32)	3.62 (1.41–8.99)
**Toilet facility**		***p‐value*** =< ***0.001***			***p‐value*** =< ***0.001***		
Improved	4483 (64.97)	2892 (64.51)	1591 (35.49)	35.10 (33.71–36.51)	4107 (91.61)	376 (8.39)	8.41 (7.63–9.26)
Unimproved	2417 (35.03)	1300 (53.79)	1117 (46.21)	45.01 (43.02–47.02)	2278 (94.25)	139 (5.75)	5.79 (4.92–6.80)
**Cooking fuel**		***p‐value*** =< ***0.001***			***p‐value*** =< ***0.001***		
Unclean	5516 (79.94)	3212 (58.23)	2304 (41.77)	40.57 (39.27–41.88)	5138 (93.15)	378 (6.85)	6.81 (6.17–7.51)
Clean	1384 (20.06)	980 (70.81)	404 (29.19)	30.70 (28.35–33.16)	1247 (90.10)	137 (9.90)	10.17 (8.70–11.86)

Abbreviations: CI, confidence interval; DBMHL, double burden of malnutrition at household level.

#### 3.1.1 Prevalence of Child Undernutrition and DBM at the Household‐Level

**Figure 2:**
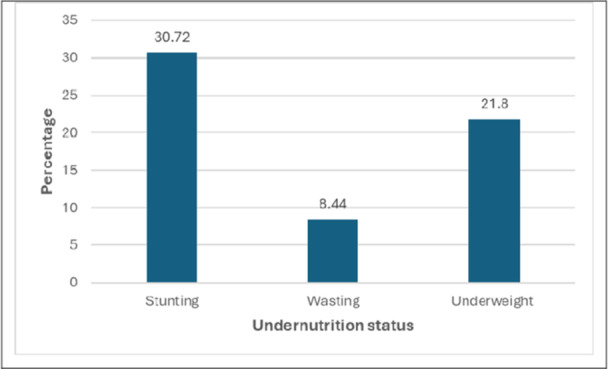
The percentage of undernutrition status of children.

Table 1 and Figure 2 also present the prevalence of child undernutrition and DBM at the household level. The overall weighted prevalence of child undernutrition was 38.54% (95% CI: 37.39%–39.70%), with stunting, wasting, and underweight accounting for 30.72%, 8.44%, and 21.80%, respectively. Undernutrition was more prevalent among children aged 24–35 months (44.99%, 95% CI: 42.37%–47.63%) compared to other age groups. Children with a birth order of 1 had a lower prevalence (36.12%, 95% CI: 34.24%–38.04%), which increased with higher birth orders. The prevalence was significantly higher among mothers (52.07%, 95% CI: 47.70%–56.40%) and fathers (50.29%, 95% CI: 47.25%–53.33%) who had no formal education, decreasing as education levels increased.

Children born to mothers aged 18 years or younger at their first birth had a higher prevalence of undernutrition (41.11%) compared to those with older mothers (34.81%). Undernutrition was more common in families without media exposure (45.11%, 95% CI: 43.16%–47.08%) and in rural areas (40.40%) compared to urban settings (33.60%). Wealthier households had lower rates of undernutrition compared to the poorest households, with the poorest (49.01%) and poorer (44.52%) quintiles showing the highest prevalence, and the richest quintile having a lower rate (24.91%, 95% CI: 22.65%–27.33%). The highest prevalence of undernutrition was observed among children from the Sylhet division (52.16%, 95% CI: 48.09%–56.20%). Additionally, children whose families used unimproved water (48.70%, 95% CI: 39.82%–57.67%), unimproved toilets (45.01%, 95% CI: 43.02%–47.02%) and unclean cooking fuel (40.57%, 95% CI: 39.27%–41.88%) had higher rates of undernutrition.

The overall prevalence of the DBM at the household level was 7.50% (95% CI: 6.90%–8.15%). This prevalence of DBM was higher in households with children aged 36–47 months (10.15%) and those with larger ( ≥ 5) birth orders (8.84%). It was also more prevalent in households with women aged 35–49 years (12.18%, 95% CI: 9.75%–15.11%) and in households where mothers had an age at first birth > 18 years (8.03%, 95% CI: 7.08%–9.10%). The highest prevalence was observed in wealthier households, with 10.22% (95% CI: 8.74%–11.92%) in richer families and 9.75% (95% CI: 8.25%–11.47%) in the richest families. Additionally, DBM was more frequent in urban areas (9.64%).

The chi‐square test at a 5% significance level revealed significant associations between child undernutrition and the DBM at the household level with factors such as children's age, birth order, wealth index, media exposure, place of residence, division, toilet facility, and cooking fuel. Maternal education, paternal education, maternal age at first birth, and drinking water sources were significantly linked to child undernutrition, while only maternal age was significantly related to the DBM (Table 1).

### 3.2 Regression Analysis

Table 2 shows the results of the binary logistic regression analysis, including adjusted odds ratio and confidence intervals for child undernutrition and the DBM at the household level. The regression analysis results revealed that children aged 12–23, 24–35, 36–47, and 48–59 months were 1.75 (AOR: 1.75, 95% CI: 1.49–2.05; *p* < 0.001), 2.01 (AOR: 2.01, 95% CI: 1.71–2.36; *p* < 0.001), 1.73 (AOR: 1.73, 95% CI: 1.46–2.04; *p* < 0.001), and 1.42 (AOR: 1.42, 95% CI: 1.20–1.67; *p* < 0.001) times more likely to be undernourished, respectively, compared to children under 12 months. The study found that children of mothers with secondary and higher education were significantly less likely to experience undernutrition, with odds ratios of 0.75 (AOR: 0.75, 95% CI: 0.60–0.93; *p* = 0.009) and 0.54 (AOR: 0.54, 95% CI: 0.40–0.71; *p* < 0.001), respectively, compared to children of uneducated mothers. Similarly, children with father who had secondary or higher education had lower odds of undernutrition. Children from the middle (AOR: 0.79, 95% CI: 0.66–0.95; *p* = 0.011), richer (AOR: 0.77, 95% CI: 0.63–0.93; *p* = 0.007), and richest (AOR: 0.53, 95% CI: 0.41–0.68; *p* < 0.001) wealth quintiles had significantly lower odds of undernutrition compared to those from the poorest quintile. Additionally, children from Khulna division had a 29% (AOR: 0.71, 95% CI: 0.57–0.89; *p* = 0.003) lower likelihood of being undernourished, while those from Sylhet division had a 43% (AOR: 1.43, 95% CI: 1.17–1.76; *p* = 0.001) higher likelihood considering Barisal as reference.

**Table 2:** Logistic regression analysis: Associated factors with child undernutrition and DBMHL with 95% CI.

Factors	Child undernutrition		DBMHL	
	AOR (95% CI)	*p* value	AOR (95% CI)	*p* value
**Children characteristics**				
**Age of child (in month)**				
< 12 (ref)	1.00		1.00	
12–23	1.75 (1.49–2.05)	**< 0.001**	1.26 (0.90–1.78)	0.180
24–35	2.01 (1.71–2.36)	**< 0.001**	2.21 (1.61–3.02)	**< 0.001**
36–47	1.73 (1.46–2.04)	**< 0.001**	2.08 (1.51–2.86)	**< 0.001**
48–59	1.42 (1.20–1.67)	**< 0.001**	1.59 (1.15–2.21)	**0.005**
**Sex of child**				
Male (ref)	1.00		1.00	
Female	0.97 (0.87–1.07)	0.504	0.90 (0.75–1.08)	0.272
**Birth order**				
1 (ref)	1.00		1.00	
2–4	0.98 (0.85–1.13)	0.779	1.14 (0.87–1.48)	0.341
≥ 5	1.06 (0.81–1.40)	0.661	1.11 (0.68–1.81)	0.676
**Parental characteristics**				
**Maternal age (in years)**				
15–24 (ref)	1.00		1.00	
25–34	1.03 (0.90–1.19)	0.631	1.74 (1.34–2.26)	**< 0.001**
35–49	1.00 (0.79–1.26)	0.972	2.58 (1.75–3.79)	**< 0.001**
**Maternal education**				
No education (ref)	1.00		1.00	
Primary	0.82 (0.67–1.01)	0.066	1.10 (0.73–1.64)	0.651
Secondary	0.75 (0.60–0.93)	**0.009**	1.09 (0.72–1.65)	0.688
Higher	0.54 (0.40–0.71)	**< 0.001**	0.81 (0.48–1.37)	0.434
**Paternal education**				
No education (ref)	1.00		1.00	
Primary	0.93 (0.79–1.08)	0.336	1.08 (0.79–1.47)	0.619
Secondary	0.76 (0.64–0.91)	**0.002**	1.13 (0.81–1.58)	0.480
Higher	0.62 (0.49–0.78)	**< 0.001**	0.96 (0.63–1.47)	0.864
**Maternal age at first birth (in years)**				
> 18 (ref)	1.00		1.00	
≤ 18	1.06 (0.94–1.19)	0.333	1.05 (0.85–1.30)	0.631
**Media exposure**				
No (ref)	1.00		1.00	
Yes	0.99 (0.88–1.12)	0.868	1.05 (0.83–1.33)	0.664
**Household characteristics**				
**Wealth index**				
Poorest (ref)	1.00		1.00	
Poorer	0.97 (0.83–1.13)	0.700	1.05 (0.75–1.48)	0.774
Middle	0.79 (0.66–0.95)	**0.011**	1.46 (1.02–2.07)	**0.037**
Richer	0.77 (0.63–0.93)	**0.007**	1.84 (1.28–2.64)	**0.001**
Richest	0.53 (0.41–0.68)	**< 0.001**	1.68 (1.08–2.62)	**0.022**
**Residence**				
Rural (ref)	1.00		1.00	
Urban	1.01 (0.89–1.14)	0.864	1.07 (0.86–1.34)	0.520
**Division**				
Barisal (ref)	1.00		1.00	
Chittagong	1.05 (0.86–1.28)	0.647	0.92 (0.64–1.31)	0.644
Dhaka	0.90 (0.73–1.12)	0.362	0.83 (0.57–1.21)	0.338
Khulna	0.71 (0.57–0.89)	**0.003**	0.63 (0.41–0.95)	**0.029**
Mymensingh	1.08 (0.88–1.33)	0.465	0.65 (0.43–0.98)	**0.038**
Rajshahi	0.90 (0.72–1.13)	0.374	0.85 (0.57–1.26)	0.414
Rangpur	0.84 (0.68–1.05)	0.127	0.90 (0.61–1.34)	0.613
Sylhet	1.43 (1.17–1.76)	**0.001**	0.71 (0.49–1.04)	0.079
**Drinking water source**				
Improved (ref)	1.00		1.00	
Unimproved	1.32 (0.92–1.88)	0.128	0.74 (0.32–1.72)	0.484
**Toilet facility**				
Improved (ref)	1.00		1.00	
Unimproved	1.05 (0.94–1.18)	0.389	0.83 (0.66–1.04)	0.110
**Cooking fuel**				
Unclean (ref)	1.00		1.00	
Clean	1.00 (0.83–1.20)	0.995	1.13 (0.84–1.51)	0.419

*Note:* Bold indicates the significance at 5% level of significance.

Abbreviations: AOR, adjusted odds ratio; CI, confidence interval; DBMHL, double burden of malnutrition at household level.

Regarding the DBM at the household level, children aged 24–35, 36–47, and 48–59 months were 2.21 (AOR: 2.21, 95% CI: 1.61–3.02; *p* < 0.001), 2.08 (AOR: 2.08, 95% CI: 1.51–2.86; *p* < 0.001) and 1.59 (AOR: 1.59, 95% CI: 1.15–2.21; *p* = 0.005) times more likely to be affected, respectively, compared to those under 12 months. Mothers aged 25–34 and 35–49 years were 1.74 (AOR: 1.74, 95% CI: 1.34–2.26; *p* < 0.001) and 2.58 (AOR: 2.58, 95% CI: 1.75–3.79; *p* < 0.001) times more likely to experience the DBM at the household level than mothers aged 15–24 years. Furthermore, households in the middle (AOR: 1.46, 95% CI: 1.02–2.07; *p* = 0.037), richer (AOR: 1.84, 95% CI: 1.28–2.64; *p* = 0.001) and richest (AOR: 1.68, 95% CI: 1.08–2.62; *p* = 0.022) wealth quintiles had higher odds of experiencing the DBM, compared to the poorest households.

### 3.3 Wealth‐Based Inequalities: Concentration Curve and Concentration Index

**Figure 3:**
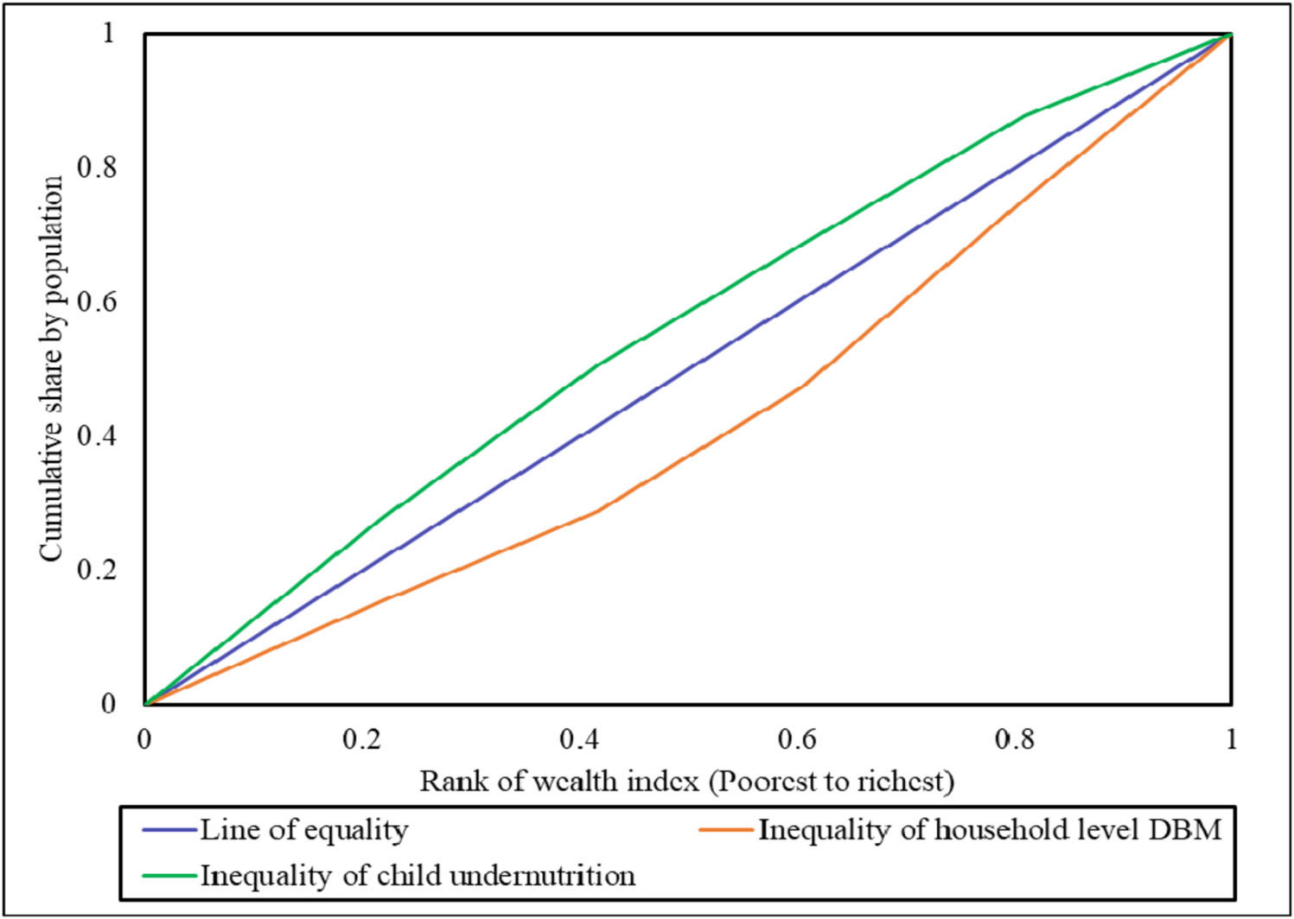
Concentration curve for child undernutrition and DBMHL based on household wealth index.

The concentration curve and concentration index revealed the inequality in child malnutrition and the DBM at the household level. The child malnutrition curve, situated above the line of equality, highlighted its disproportionate prevalence among families in lower wealth groups. Conversely, the curve for the DBM lay below the 45‐degree line, indicating that it was more concentrated in wealthier households (Figure 3).

Table 3 presents the decomposition results, including elasticity, concentration index, absolute contribution, and percentage contribution, shedding light on how various socio‐economic factors contribute to inequalities in child malnutrition and the DBM at the household level. The decomposition of the concentration index (CIX) illustrates the role of different explanatory variables in either reducing or amplifying the observed inequalities, with a negative contribution indicating a reduction and a positive contribution indicating an increase in inequality.

**Table 3:** Decomposition of the concentration indexes (CIXs) of child undernutrition and DBMHL to determine the contributions of sociodemographic factors on each CIX.

Factors	Undernutrition overall CIX =−0.191 (*p* < 0.001)				DBMHL overall CIX = 0.163 (*p* < 0.001)			
	Elasticity	CIX	Absolute	% CIX	Elasticity	CIX	Absolute	% CIX
**Children characteristics**								
**Age of child (in months)**								
< 12 (ref)								
12–23	0.068	0.011	0.001	−0.40	0.056	0.011	0.001	0.38
24–35	0.086	0.009	0.001	−0.38	0.148	0.009	0.001	0.78
36–47	0.062	0.028	0.002	−0.92	0.142	0.028	0.004	2.48
48–59	0.044	−0.030	−0.001	0.70	0.106	−0.030	−0.003	−1.98
Sub‐total		0.018	0.003	−1.00		0.018	0.003	1.66
**Sex of child**								
**Male (ref)**								
Female	−0.008	−0.003	0.000	−0.01	−0.032	−0.003	0.000	0.06
Sub‐total		−0.003	0.000	−0.01		−0.003	0.000	0.06
**Birth order**								
**1 (ref)**								
2–4	−0.006	−0.061	0.000	−0.19	0.013	−0.061	−0.001	−0.47
≥ 5	0.002	−0.315	−0.001	0.38	−0.003	−0.315	0.001	0.64
Sub‐total		−0.376	−0.001	0.19		−0.376	0.000	0.17
**Parental characteristics**								
**Maternal age (in years)**								
15–24 (ref)								
25–34	−0.003	0.031	0.000	0.04	0.243	0.031	0.008	4.65
35–49	−0.006	−0.012	0.000	−0.04	0.070	−0.012	−0.001	−0.52
Sub‐total		0.019	0.000	0.00		0.019	0.007	4.14
**Maternal education**								
**No education (ref)**								
Primary	−0.029	−0.337	0.010	−5.08	0.049	−0.337	−0.017	−10.21
Secondary	−0.083	0.097	−0.008	4.20	−0.024	0.097	−0.002	−1.43
Higher	−0.057	0.554	−0.031	16.42	−0.060	0.554	−0.033	−20.40
Sub‐total		0.314	−0.029	15.54		0.314	−0.052	−32.05
**Paternal education**								
**No education (ref)**								
Primary	−0.033	−0.299	0.010	−5.13	0.034	−0.299	−0.010	−6.22
Secondary	−0.056	0.214	−0.012	6.28	0.085	0.214	0.018	11.26
Higher	−0.045	0.556	−0.025	13.19	0.030	0.556	0.017	10.16
Sub‐total		0.471	−0.027	14.34		0.471	0.025	15.20
**Maternal age at first birth**								
> 18 (ref)								
≤ 18	0.013	−0.256	−0.003	1.79	0.019	−0.256	−0.005	−2.96
Sub‐total		−0.256	−0.003	1.79		−0.256	−0.005	−2.96
**Maternal media exposure**								
No (ref)								
Yes	−0.015	0.587	−0.008	4.4	0.006	0.586	0.004	2.27
Sub‐total		0.587	−0.00	4.43		0.586	0.004	2.27
**Household characteristics**								
Wealth index								
**Poorest (ref)**								
Poorer	−0.007	−0.460	0.003	−1.56	−0.009	−0.460	0.004	2.66
Middle	−0.028	0.026	−0.001	0.38	0.051	0.026	0.001	0.81
Richer	−0.028	0.520	−0.015	7.60	0.111	0.520	0.058	35.64
Richest	−0.067	1.000	−0.067	34.91	0.088	1.000	0.088	54.38
Sub‐total		1.086	−0.080	41.33		1.086	0.151	93.49
**Residence**								
**Rural (ref)**								
Urban	0.002	0.542	0.001	−0.56	0.031	0.542	0.017	10.33
Sub‐total		0.542	0.001	−0.56		0.542	0.017	10.33
**Division**								
**Barisal (ref)**								
Chittagong	−0.001	0.075	0.000	0.03	−0.007	0.075	−0.001	−0.33
Dhaka	−0.027	0.346	−0.009	4.81	−0.057	0.346	−0.020	−12.22
Khulna	−0.020	0.031	−0.001	0.33	−0.039	0.031	−0.001	−0.75
Mymensingh	−0.001	−0.235	0.000	−0.18	−0.034	−0.235	0.008	4.92
Rajshahi	−0.005	−0.092	0.001	−0.24	0.002	−0.092	0.000	−0.14
Rangpur	−0.012	−0.297	0.004	−1.84	−0.012	−0.296	0.004	2.20
Sylhet	0.017	−0.134	−0.002	1.17	−0.021	−0.134	0.003	1.74
Sub‐total		−0.30	−0.008	4.08		−0.306	−0.007	−4.57
**Drinking water source**								
**Improved (ref)**								
Unimproved	0.003	−0.408	−0.001	0.56	−0.007	−0.408	0.003	1.83
Sub‐total		−0.408	−0.001	0.56		−0.408	0.003	1.83
**Toilet facility**								
**Improved (ref)**								
Unimproved	0.014	−0.503	−0.007	3.60	−0.060	−0.503	0.030	18.50
Sub‐total		−0.503	−0.007	3.60		−0.503	0.030	18.50
**Cooking fuel**								
**Unclean (ref)**								
Clean	0.010	0.779	0.008	−3.91	0.023	0.779	0.018	10.78
Sub‐total		0.779	0.008	−3.91		0.779	0.018	10.78
Explained			−0.152	80.38			0.194	118.85
Residual			−0.039	19.62			−0.031	−18.85

Abbreviations: CIX, concentration index; DBMHL, double burden of malnutrition at household level; *p*, *p* value.

The overall wealth‐based CIX for child undernutrition was ‐0.191 (*p* < 0.001), indicating that undernourished children were primarily concentrated in lower wealth quintiles. This study identified that maternal and paternal education, household wealth index and maternal media exposure contributed the most to the wealth based inequality in child undernutrition. The wealth‐based CIX for the DBM at the household level was 0.163 (*p* < 0.001), indicating that the DBM was more concentrated in higher wealth quintiles. Similar to child undernutrition, maternal and paternal education, wealth index, and toilet facility were the highest contributors to the observed inequality in DBM.

**Figure 4:**
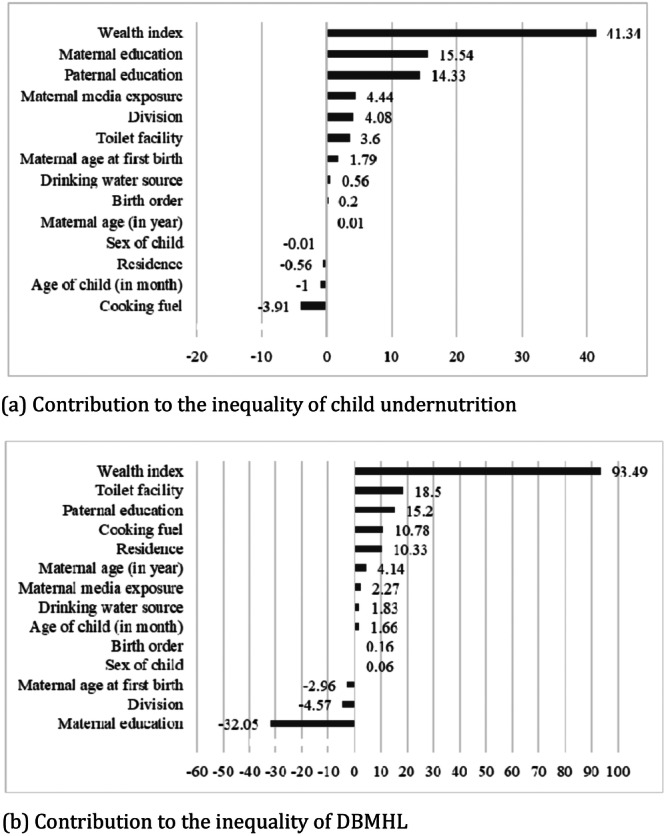
Percentage contributions of socio‐economic factors in concentration indexes of child undernutrition and DBMHL. (a) Contribution to the inequality of child undernutrition. (b) Contribution to the inequality of DBMHL.

Figure 4 illustrates the percentage contributions of socio‐economic factors to the concentration indexes of child undernutrition and the double burden of malnutrition at household level (DBMHL). For child undernutrition, the wealth index contributes the highest at 41.34%, followed by maternal education at 15.54%, and paternal education at 14.33%, highlighting significant disparities among poorer and less‐educated groups. For DBMHL, the wealth index also plays a dominant role, contributing 93.49%, while toilet facility accounts for 18.50%, and paternal education contributes 15.20%, emphasizing that poorer households disproportionately face the burden.

## 4 Discussion

This study analyses the BDHS 2017–18 data to examine the prevalence, determinants, and wealth‐based inequalities of child undernutrition, along with the DBM at the household level in Bangladesh. The findings reveal that the overall prevalence of child undernutrition and household‐level DBM stood at 38.54% and 7.50%, respectively. While child undernutrition was less prevalent in India compared to Bangladesh, the DBM at the household level was notably higher [31]. However, the prevalence of DBM at the household level was found to be higher in a study using the 2017–18 BDHS data compared to a study conducted with the 2014 BDHS data [43]. This study identified age, wealth index, and geographic division as key factors influencing both child undernutrition and household‐level DBM, with maternal and paternal education specifically impacting child undernutrition, and maternal age affecting the double burden. Notable wealth‐based inequalities were also observed in both conditions.

This study observed that the child undernutrition prevalence was slightly lower than that reported in India [31], while the DBM at the household level was less frequent in our country compared to the data from 2014 and findings from other nations, such as Pakistan, India, and the broader South and South Asian regions [41]. Notably, an increase in children's age was connected with a rising trend in the DBM at the household level, aligning with results from a research in India [31]. Interestingly, we identified an inverse relationship between child undernutrition and the DBM with respect to wealth index: while the prevalence of child undernutrition decreased with increasing wealth status, the DBM at the household level showed a rising trend, corroborated by studies conducted in Bangladesh [6], India [31], and sub‐Saharan Africa [1]. Additionally, the DBM was more prevalent in urban regions compared to rural ones, mirroring patterns observed in India [31]. Lastly, households with unimproved toilet facilities had a higher proportion of undernourished children than those with improved facilities, a finding previously reported in studies utilising BDHS 2004 and BDHS 2014 data in Bangladesh [6]. Older children are more prone to undernutrition and the DBM, as highlighted by studies in Bangladesh [28], Ethiopia [29], Pakistan, and Myanmar [27]. In developing countries like Bangladesh, the rising nutritional demands of growing children often exceed household capacities, contributing to these challenges.

Maternal age significantly impacts DBM at the household level but not child undernutrition. As maternal age increases, so does the likelihood of a household experiencing this burden, aligning with studies from Bangladesh [27, 30] and India [31]. Older mothers often have a slower metabolism and consume more calories, which increases the likelihood of being overweight or obese compared to younger mothers [16]. This trend emphasizes the critical role of maternal health in influencing child health and contributing to the DBM at the household level [46]. This study found that higher maternal and paternal education had a limited impact on child undernutrition and no significant effect on the DBM, aligning with prior research in Bangladesh [28]. Educated parents likely possess greater knowledge of health and nutrition, enabling improved care for their children and themselves [47]. Children from wealthier households tend to have a lower risk of undernutrition; however, the opposite was observed for the DBM at the household level. Several studies have established a link between household wealth and the DBM, highlighting the complex relationship between socioeconomic status and this dual challenge [27, 29, 41, 42, 48]. Higher income levels often lead to greater access to energy‐dense, unhealthy foods, which can contribute to adult obesity. Meanwhile, childhood undernutrition remains prevalent due to insufficient focus on children's biological needs, caregiving practices, diet quality, intra‐household food distribution, and micronutrient intake [49]. As a result, despite increased calorie consumption, these households overlook essential factors for enhancing child nutrition, thereby sustaining the DBM [43]. Furthermore, in low‐ and middle‐income countries (LMICs), urbanization, income growth, and trade liberalization have driven private investment in the food sector, accelerating dietary transitions. This reflects a “nutrition transition” paradox, where rising wealth increases access to ultra‐processed foods which leads to maternal overnutrition and child undernutrition within the same households [37].

This study underscores the impact of geographical divisions on child undernutrition in Bangladesh, with children from Sylhet showing higher rates of undernutrition and those from Khulna lower rates. The findings also highlight regional differences in the DBM at the household level, with Khulna and Mymensingh showing lower risks compared to Barisal, suggesting that lifestyle factors influenced by location play a significant role in the health outcomes of children. This study found that children from Sylhet were more likely to be undernourished compared to those from Barisal, while children from Khulna had lower undernutrition risks, similar to previous research on child malnutrition in Bangladesh [28]. Additionally, the DBM was less common in Khulna and Mymensingh compared to Barisal, aligning with earlier studies [48]. Regional variations in child health outcomes can be attributed to varying lifestyle factors, such as food habits and access to healthcare, across different geographical locations in Bangladesh [50].

## 5 Wealth‐Based Inequalities

This study highlights wealth‐based inequalities in child undernutrition and the DBM at the household level in Bangladesh. Child undernutrition was predominantly found in lower wealth groups, aligning with studies from Bangladesh [6] and India [44], while wealthier households experienced the DBM, consistent with findings in Bangladesh [48] and India [31]. In the South Asian region, such contrast situation (overweight mother with undernourished child) may arise from practices more common in middle‐ and high‐income households—such as excessive intake of processed energy‐dense foods, soft drink consumption, and lack of physical activity, which may adversely affect mothers and children in opposite ways [27]. The decomposition analysis identified wealth status as the key factor contributing to these inequalities. Maternal education was found to reduce the overall obtained inequality in DBM but contribute to the inequality in child undernutrition, while paternal education enhanced the disparities in both. Higher maternal education improves health knowledge, enabling wealthier mothers to provide healthier diets for their children, while educated but economically disadvantaged mothers may still face limited access [47]. By addressing maternal overweight/obesity, the prevalence of DBM can be reduced, since DBM meets this criterion [37]. Explicitly, education can play a protective role against overweight/obesity, with a stronger effect in affluent households where overweight/obesity is more prevalent. Thus, maternal education may increase the wealth‐based gap in child undernutrition while reducing it in DBM. Other factors, such as place of residence, toilet facilities, and cooking fuel, also enhanced the wealth‐based inequality in DBM, while maternal media exposure enhanced this inequality in child undernutrition. Addressing wealth disparities through health education programmes and promoting balanced nutrition in low‐income families can improve child health outcomes [30, 46]. Energy balancing programmes can benefit individuals with obesity, particularly in higher wealth groups, by promoting physical activity and reducing calorie intake [51]. Access to educational programmes on obesity prevention should be provided to communities, companies, and schools [52]. Conversely, families with limited resources in Bangladesh are more likely to have malnourished children due to their inability to afford nutritious meals, highlighting the need for a focus on accessible, healthy diets rich in fruits and vegetables [30, 46].

## 6 Policy Implications

Policymakers should design targeted nutritional programmes for low‐income families, raise awareness about child malnutrition, tackle the double burden in wealthier families, ensure equal healthcare access, and improve social protection. Community‐based initiatives, nutritional education in schools, and effective monitoring are crucial steps, with ongoing research and public‐private partnerships essential for addressing disparities and improving maternal and child health.

## 7 Strengths and Limitations

This study's strength lies in its use of nationally representative data and robust estimation methods, enabling the findings to be generalised to a wider population. The decomposition approach offers valuable insights into how child‐related factors, parental characteristics, and household factors contribute to child undernutrition and the DBM among child‐mother pairs in Bangladesh. However, the study has some limitations, including its cross‐sectional design, which avoids the establishment of causal associations, and the lack of consideration of regional factors that could influence the association between socioeconomic status and child malnutrition outcomes. Future research should explore the effects of additional structural, contextual, and regional factors, such as community poverty, access to healthcare, and local cultural influences on child health.

## 8 Conclusion

This study highlights significant disparities in nutritional outcomes related to socio‐economic status in Bangladesh, emphasising the need for targeted interventions to address both undernutrition and overnutrition within vulnerable populations. Key factors contributing to malnutrition inequalities include wealth status, education levels of women and their partners, and access to toilet facilities. Younger children are at a higher risk of undernutrition and DBM, while factors like higher child age, maternal education, paternal education, and better wealth status are linked to lower risks. Conversely, older children, older mothers, and higher wealth indices increase the risk of DBM. To mitigate these issues, urgent interventions are needed, particularly targeting families from higher socio‐economic statuses.

## Author Contributions

Conceptualization: Sumaya Sultana, Khondokar Naymul Islam, and Benojir Ahammed. Data curation: Sumaya Sultana, and Khondokar Naymul Islam. Formal analysis: Sumaya Sultana, and Khondokar Naymul Islam. Investigation: Benojir Ahammed, Syed Afroz Keramat. Methodology: Sumaya Sultana, and Khondokar Naymul Islam. Resources: Sumaya Sultana Software: Sumaya Sultana, and Khondokar Naymul Islam. Supervision: Benojir Ahammed, Syed Afroz Keramat. Validation: Sumaya Sultana, Khondokar Naymul Islam, and Benojir Ahammed. Visualization: Sumaya Sultana, Khondokar Naymul Islam, and Benojir Ahammed. Writing – original draft: Sumaya Sultana, Khondokar Naymul Islam, and Benojir Ahammed. Writing – review and editing: Benojir Ahammed, and Syed Afroz Keramat. All authors read and approved the final version of the manuscript.

## Disclosure

The lead author Benojir Ahammed affirms that this manuscript is an honest, accurate, and transparent account of the study being reported; that no important aspects of the study have been omitted; and that any discrepancies from the study as planned (and, if relevant, registered) have been explained.

## Ethics Statement

This study used publicly available and fully anonymised data; therefore, no additional ethical approval or participant consent was required.

## Conflicts of Interest

The authors declare no conflicts of interest.

## Data Availability

The data used in this study are publicly available from the Demographic and Health Surveys (DHS) Program. The Bangladesh Demographic and Health Survey (BDHS) datasets can be accessed upon reasonable request from the DHS Program website: https://dhsprogram.com/data/. Researchers must register for access and specify the purpose of data use. The DHS Program grants access for academic and non‐commercial research purposes.
